# The Cellular Respiration of Endometrial Biopsies from Patients with Various Forms of Endometriosis

**DOI:** 10.3390/ijms25073680

**Published:** 2024-03-26

**Authors:** Konstantin A. Toniyan, Artyom A. Malkov, Nikolay S. Biryukov, Elena Yu. Gorbacheva, Valery V. Boyarintsev, Irina V. Ogneva

**Affiliations:** 1 Cell Biophysics Laboratory, State Scientific Center of the Russian Federation Institute of Biomedical Problems of the Russian Academy of Sciences, 123007 Moscow, Russia; ktoniyan@mail.ru (K.A.T.); malkov.aa.2003@yandex.ru (A.A.M.); biryukovns@gmail.com (N.S.B.); elenagorbacheva22@gmail.com (E.Y.G.); 2 Gynecology Department, FGBU KB1 (Volynskaya) UDP RF, 121352 Moscow, Russia; 3 Medical and Biological Physics Department, I.M. Sechenov First Moscow State Medical University (Sechenov University), 8-2 Trubetskaya Street, 119991 Moscow, Russia; 4 Emergency and Extreme Medicine Department, FGBU DPO CGMA UDP RF, 121359 Moscow, Russia; wpx@mail.ru

**Keywords:** endometriosis, adenomyosis, OXPHOS, glycolysis, macrolides

## Abstract

Endometriosis is one of the leading pathologies of the reproductive system of women of fertile age, which shows changes in cell metabolism in the lesions. We conducted a study of the cellular respiration according to the polarography and the mRNA content of the main metabolic proteins using qRT-PCR of intraoperative endometrial biopsies from patients in the control group and with different localizations of endometriosis (adenomyosis, endometrioma, pelvic peritoneum). In biopsy samples of patients with endometriomas and pelvic peritoneum endometriotic lesions, the rate of oxygen absorption was significantly reduced, and, moreover, in the extragenital case, there was a shift to succinate utilization. The mRNA content of the cytochrome c, cytochrome c oxidase, and ATP synthase was also reduced, but hexokinase HK2 as well as pyruvate kinase were significantly higher than in the control. These oxidative phosphorylation and gene expression profiles suggest the Warburg effect and a shift in metabolism toward glycolysis. For adenomyosis, on the contrary, cellular respiration was significantly higher than in the control group due to the terminal region of the respiratory chain, ATP synthase, and its mRNA was increased as well. These data allow us to suggest that the therapeutic strategies of endometriosis based on modulation energy metabolism should take lesion localization into account.

## 1. Introduction

Endometriosis is a widespread gynecological disease that mainly affects women of reproductive age. In addition to pain, endometriosis often leads to decreased fertility. This pathology is characterized by the appearance of an ectopic focus of endometrial-like tissue outside the epithelial layer of the uterine cavity [1]. Depending on the location, a distinction is made between genital (adenomyosis—growth of the endometrium into the muscular layer of the uterus and endometriotic ovarian cysts) and extragenital (most often, lesions are found on the peritoneal mesothelium, but there are also cases of localization far beyond the abdominal cavity, in particular in the lungs [2,3] and nasolacrimal channel [4,5]).

Despite the long duration of the study, there is no consensus on the etiology of endometriosis, and the pathogenesis of endometriosis is multifactorial [6]. The appearance and growth of lesions is most often associated with hormonal imbalance; retrograde menstruation [7,8,9,10,11,12]; and even with errors in the migration of mesodermal germ layer cells in embryonic development [13,14,15], which may be associated with aberrant regulation of the proteins of the Wnt signaling pathway [16].

The complex and ambiguous pathogenesis of endometriosis does not allow us to develop a unified approach to its treatment. In most cases, treatment involves organ-conserving surgery, but, nevertheless, it increases the risks of a mechanical decrease in ovarian reserve and possible postoperative complications. However, for endometriomas, sclerotherapy has been used over the past few decades [17] using various agents —methotrexate [18], tetracycline [19] and, most often, ethanol [20]—with transvaginal access [17,21]. But in the case of adenomyosis, with repeated bleeding, radical surgical treatment is performed, i.e., a hysterectomy [22], the consequences of which can also reduce the quality of life of patients. An accepted therapeutic treatment strategy is the use of hormonal drugs, which is accompanied by a large number of restrictions [23] and is, in some cases, contraindicated, especially if the patient is planning a pregnancy. Therefore, the search for new strategies for the therapeutic treatment of endometriosis remains relevant.

Recently, the attention of researchers has been attracted to approaches to non-hormonal therapy based on modulation of energy metabolism [24]. In ectopic endometrial foci, cells are in a hypoxic environment [25], but nevertheless survive, probably due to a switch from oxidative phosphorylation to glycolysis, as shown in a nonhuman primate model [26] as well as in peritoneal mesothelial cells with endometriotic lesions [27]. In addition, endometrial cells that have lost their eutopic localization avoid anoikis [28,29], possibly due to overexpression of the anti-apoptotic protein Bcl-2 [30]. Bcl-2 inhibits the pathway of apoptosis, which is initiated by the increase in the cytochrome c in the cytoplasm [31,32].

The complex I of the mitochondrial respiratory chain is one of the main sources of reactive oxygen species in the cell [33]. Therefore, a decrease in the cellular respiration should be accompanied by a decrease in ROS production. However, ROS levels in patients with endometriosis have been shown to be higher in both serum and follicular fluid compared to controls [34], as well as markers of lipid peroxidation [35]. Our study of biopsy samples from patients with adenomyosis showed that the content and expression level of terminal complexes of the respiratory chain were higher than in the control, suggesting the effectiveness of the use of macrolide antibiotics as therapeutic agents [36]. Moreover, the use of oxidative phosphorylation (OXPHOS) inhibitors reduces the proliferation of endometriosis cells of the 12Z line [37].

Thus, on the one hand, a number of results indicate a decrease in OXPHOS and a switch to glycolysis in the foci in endometriosis [26,27]. On the other hand, in some cases, inhibitors of oxidative phosphorylation prevent the growth of endometrial cells [37].

These results seem somewhat contradictory, probably because some of these results were obtained in situ from human endometriotic lesions of various localizations or not in humans. That is why we decided to conduct a study to determine, ex vivo, the intensity of the mitochondrial respiration in endometrial biopsies of patients in the control group (C) and patients with endometriosis of various localizations: adenomyosis (AM), genital endometriosis (GE, endometrioid cysts of the ovaries, fallopian tubes, posterior surface of the cervix). and extragenital endometriosis (EGE, endometriotic lesions on the pelvic peritoneum). In addition, we determined the expression of key proteins of the mitochondrial respiratory chain and enzymes that catalyze irreversible reactions of glycolysis. The obtained results show that adenomyosis is characterized by an increase in the oxygen consumption rate, while the GE and EGE groups show decreases in the rate of oxygen uptake and overexpression of pyruvate kinase M1/2, which is characteristic of a switch to glycolysis.

## 2. Results

### 2.1. The OXPHOS Level in Endometrial Biopsies

**Figure 1:**
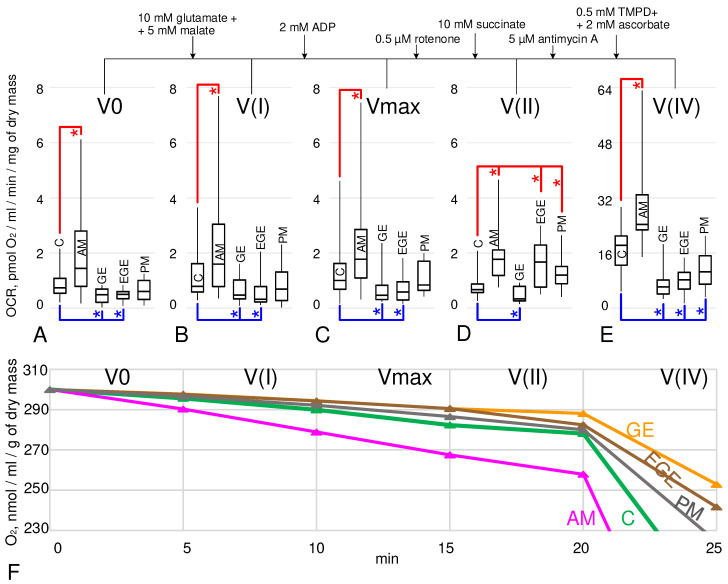
Oxygen consumption rates (OCRs) of endometrial biopsies in patients without and with endometriosis of various locations. C, control group; AM, adenomyosis; GE, genital endometriosis; EGE, extragenital endometriosis; PM, postmenopause. A detailed description of the groups is presented in the experimental design in the Materials and Methods section. The OCR was measured sequentially, starting with V0—basal OCR (**A**). Then, V(I) (**B**)—OCR after 10 mM glutamate + addition of 5 mM malate. Then, Vmax (**C**)—maximal OCR after addition of 2 mM ADP. Next, V(II) (**D**)—OCR after subsequently adding 0.5 µM rotenone (complex I inhibitor) and 10 mM succinate (complex II substrate). Finally, V(IV) (**E**)—OCR after subsequently adding 5 µM antimycin A (complex III inhibitor), 0.5 mM TMPD + 2 mM ascorbate (artificial complex IV substrates). Full normalized curves of the oxygen consumption for each group (**F**) are presented in different colors: C—green; AM—pink; GE—orange; EGE—brown; PM—grey. *—*p* < 0.05 in comparison with the control group C, with additional marking of decreases in blue color and increases in red color.

**Figure 2:**
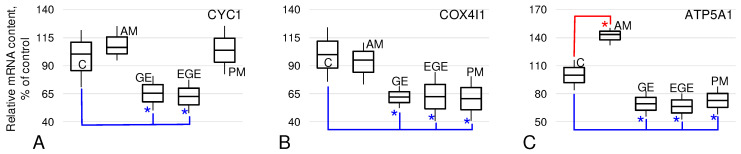
The relative mRNA content of the proteins formed the main complexes of the respiratory chain in endometrial biopsies from patients without and with endometriosis of various localizations. As above, C, control group; AM, adenomyosis; GE, genital endometriosis; EGE, extragenital endometriosis; PM, postmenopause. A detailed description of the groups is presented in the experimental design section of the Materials and Methods. (**A**)—CYC1, cytochrome c-1. (**B**)—COX4I1, cytochrome c oxidase subunit IV isoform 1. (**C**)—ATP5A1, ATP synthase, H+ transporting, mitochondrial F1 complex, alpha subunit 1. *—*p* < 0.05 in comparison with the control group C with additional marking of decreases in blue color and increases in red color.

The basal rate of oxygen uptake, V0 (Figure 1A), in the AM group was higher by 95% (*p* < 0.05); in the GE and EGE groups, it was lower by 35% and 32% (*p* < 0.05), respectively; and in the PM group, it was not changed compared to group C. Similar relationships occurred when measuring the oxygen consumption rate (OCR) with the addition of the substrates complex I–V(I) (Figure 1B) and 2 mM ADP–Vmax (Figure 1C).

After complex I inhibition and succinate addition, V(II) in the group AM was higher (Figure 1D) than in group C by 164% (*p* < 0.05). In group GE, V(II) was decreased by 49% (*p* < 0.05). But, for groups EGE and PM, V(II) was significantly higher in comparison with group C by 149% and 79% (*p* < 0.05), respectively.

However, after inhibition of complex III, OCR V(IV) (Figure 1E) in the AM group was higher by 34% (*p* < 0.05), and in the GE, EGE, and PM groups it was lower by 66%, 55%, and 42% (*p* < 0.05), respectively, compared to the control group.

By analyzing the normalized curves (Figure 1F) for each group, we can assume that in the case of adenomyosis (pink curve), oxygen uptake by mitochondria in ex vivo conditions is faster than in group C (green curve), but for the groups GE (orange curve) and EGE (brown curve), it is slower.

### 2.2. Gene Expression of the Main Complexes, Involving in the OXPHOS and Glycolysis

The relative content of cytochrome c mRNA, CYC1 (Figure 2A), in the AM and PM groups did not differ from the control level. In the GE and EGE groups, it was lower than the level of group C by 35% and 37% (*p* < 0.05), respectively.

The relative content of cytochrome c-oxidase mRNA (Figure 2B) in the AM group was at the level of group C; in the GE, EGE, and PM groups, it was lower than the control by 38%, 37%, and 40% (*p* < 0.05), respectively.

The relative content of ATP5A1, one of the subunits of ATP synthase (Figure 2C), in the AM group exceeded the control level by 43% (*p* < 0.05). In the GE, EGE, and PM groups, it was reduced relative to group C by 31%, 34%, and 28% (*p* < 0.05), respectively.

**Figure 3:**
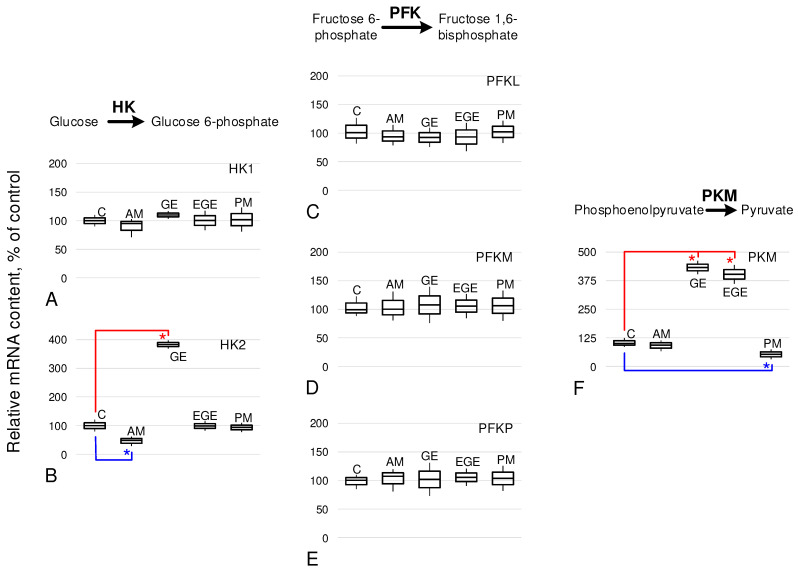
The relative mRNA content of the main metabolic proteins catalyzed irreversible reactions of the glucose utilization in endometrial biopsies from patients without and with endometriosis of various localizations. As above, C, control group; AM, adenomyosis; GE, genital endometriosis; EGE, extragenital endometriosis; PM, postmenopause. A detailed description of the groups is presented in the experimental design section of the Materials and Methods. (**A**) HK1: hexokinase 1. (**B**) HK2: hexokinase 2. (**C**–**E**) PFKL, PFKM, PFKP—phosphofructokinase, liver type, and muscle type and platelet, respectively. (**F**) PKM: pyruvate kinase M1/2. *—*p* < 0.05 in comparison with the control group C with additional marking of decreases in blue color and increases in red color.

The relative content of hexokinase HK1 (Figure 3A) was at the same level in all study groups. At the same time, for HK2 (Figure 3B), there was a decrease in the AM group by 52% (*p* < 0.05); in the GE group, there was an increase of 283% (*p* < 0.05) relative to group C. In the EGE and PM groups, the relative content of HK2 mRNA did not change.

The relative content of mRNA of the various phosphofructokinase isoforms (PFKL, PFKM and PFKP) was not changed in any of the study groups compared to the controls (Figure 3C–E).

The relative abundance of pyruvate kinase M1/2 (PKM) mRNA (Figure 3F) in the AM group was at the control level. In the GE and EGE groups, it was higher by 332% and 302% (*p* < 0.05), respectively; in the PM group, it was lower by 48% (*p* < 0.05) compared to group C.

## 3. Discussion

Today, endometriosis is one of the main gynecological pathologies of women of reproductive age, and often leads to infertility and a significant decrease in the quality of life due to chronic pelvic pain [6,7,8,38]. If the patient still plans to implement reproductive function, surgical treatment, as well as the use of hormonal drugs, may have limitations or even be contraindicated. Therefore, the search for new approaches to the treatment of endometriosis of various localizations continues to be relevant. One possible way is to influence the metabolism of endometriosis foci [24,39]. Therefore, we decided to study the final point in energy production in the metabolism of most types of cells—the rate of oxygen absorption during oxidative phosphorylation—in endometrial biopsies of patients without endometriosis and those with endometriosis of various localizations.

The obtained results indicate that the rate of oxygen uptake (Figure 1) by cells of endometriotic lesions from the pelvic peritoneum (group EGE), as well as from ovarian endometriotic cysts (GE), was significantly reduced compared to endometrial cells of patients in the control group (group C). However, if, for the GE group, this decrease took place in all parts of the respiratory chain, then in the EGE group, the rate of oxygen absorption increased with the addition of succinate, which may indicate a shift in metabolism towards its preferential utilization rather than malate. A similar transition to the utilization of succinate by mitochondria was noted during the induction of cerebral ischemia in mice [40], so it can be assumed that the effect we observed is also associated with the development of these foci under hypoxic conditions [25].

In general, the decrease in OCR in the GE and EGE groups correlated with a decrease in the relative mRNA content of genes encoding the main terminal complexes of the mitochondrial respiratory chain—cytochrome c, cytochrome c oxidase, and ATP synthase (Figure 2). However, the mRNA content of enzymes encoding the first rate-limiting stages of glucose utilization (irreversible reactions), hexokinase HK1 and phosphofructokinase (PFKL, PFKM, and PFKP), did not differ from the control (Figure 3A,C–E). However, it should be noted that the relative content of HK2 hexokinase mRNA in the GE group was increased (Figure 3B), which was previously noted in endometriomas [41]. An increase in HK2 in the case of endometriosis may be associated with hormonal imbalance, since an increase in HK2 occurs with an increase in progesterone, which is associated with the development of endometrioid cysts [42].

At the same time, the relative content of pyruvate kinase M1/2 (PKM) mRNA in endometriosis biopsies of the GE and EGE groups was significantly higher than in the control group (Figure 3F). An increase in PKM expression leads to the development of the Warburg effect, characteristic of malignant cells, which, even in the presence of oxygen, switch to glycolysis to obtain energy [43,44]. An increase in PKM can lead to its accumulation in the intercellular space and induce cell migration through p-Tyr42 RhoA-mediated superoxide generation [45], leading to the formation of extragenital lesions. In addition, the RhoA-dependent pathway may also be involved in an increase in the expression of a number of cytoskeletal proteins associated with an increase in migration potential, which we observed depending on the distance from the site of eutopic localization [46]. The results we obtained in this study are fully consistent with data on a decrease in OXPHOS in human peritoneal mesothelial and endometrial cells of nonhuman primates with endometriosis [26,27] and a transition to aerobic glycolysis [24,39,47]. Therefore, it could be suggested, after [24], that glycolysis inhibitors are good candidates for therapeutic treatment of endometriosis, but especially for endometriomas and extragenital endometriosis.

However, for adenomyosis, we obtained completely different results. The maximum rate of oxygen uptake by endometrial cells of patients in the AM group was significantly higher than in the control group C (Figure 1C). Substrate inhibitory assay data showed that V(IV) was also increased (Figure 1E), suggesting a leading role for ATP synthase in upregulating OCR. For this group, we also determined the mRNA content of the cytochrome c gene, cytochrome c oxidase, and ATP synthase. As in our previous study [36], CYC1 and COX4I1 mRNA did not differ from the control group, while for ATP synthase, we observed a significant increase (Figure 2). The relative content of HK2 mRNA in the AM group was significantly lower than in the control (Figure 3B), which can be considered as a decrease in the contribution of glycolysis to energy production by endometrial cells and a decrease in the probability of possible endometrial hyperplasia [48], which causes particular concern in adenomyosis. The increase in OCR in adenomyosis makes drugs that are inhibitors of cellular respiration promising for therapeutic treatment, as was shown in an in vitro study of endometriosis cells of the 12Z line [37]. According to our ex vivo data, this increase is due to terminal complexes of the respiratory chain. Therefore, we can carefully suggest that targeted inhibition of ATP synthase, for example, macrolide antibiotics such as josamycin, etc., may be effective enough and have fewer side effects.

Above, we compared patients of reproductive age with endometriosis of various localizations and a control group. The control group consisted of patients who, at the time of the study, had no current or history of indications of endometriosis. However, their average age was 37 ± 3 years, which does not exclude the appearance of signs of the disease in some of them in the future. Therefore, we tried to create another quasi-control group of PM, postmenopausal patients who had never had endometriosis. Interestingly, during substrate–inhibitor analysis, we saw some shift toward succinate utilization: The oxygen consumption rate V(II) in the PM group was significantly higher than in group C (Figure 1D), while V(IV) was significantly lower (Figure 1E), which was also the case in the group with extragenital endometriosis. But, unlike the EGE group, this shift was balanced, since the maximum OCR of biopsies from patients in the PM group did not differ from group C (Figure 1C). Moreover, in these patients, the relative content of mRNA was reduced not only for components involved in OXPHOS (with the exception of CYC1), but also for the main enzyme of glycolysis, pyruvate kinase M1/2 (Figure 2 and Figure 3). Postmenopause is the age of suspicion for a number of cancers, in particular endometrial cancer [49]. Patients included in the statistics of the PM group did not have any histological signs of endometrial malignancy, and the relative content of cytochrome c mRNA was the same as in patients of fertile age in the control group (Figure 2A), which gives grounds for us to very cautiously assume the maintenance of a normal mitochondria-dependent apoptosis pathway in these patients.

## 4. Materials and Methods

### 4.1. Experimental Design

This cohort study used biomaterial from 91 patients treated in the gynecology department at Gynecology Department Clinical Hospital #1 (Volynskaya, Moscow, Russia) in 2022–2023. Examination of patients, pre-surgery preparation, surgery, and management in the postsurgery period were carried out in accordance with the Order of the Ministry of Health of the Russian Federation of 1 November 2012, # 572n, with changes and additions on 17 January 2014, 11 June 2015, and 12 January 2016. All patients were in stable clinical condition with no clinical, microbiological, or laboratory evidence of infection, encephalopathy, renal failure, or comorbidities, including heart failure, pulmonary disease, malignancy, or diabetes mellitus. Written informed consent was obtained from each patient prior to participation in the study. The study design and procedures were approved by the Biomedicine Ethics Committee of the Institute of Biomedical Problems, Russian Academy of Sciences (Physiology Section of the Russian Bioethics Committee, Russian Federation National Commission for UNESCO, Permit #523/MSK/09/26/19) and conformed to the Declaration of Helsinki.

**Figure 4:**
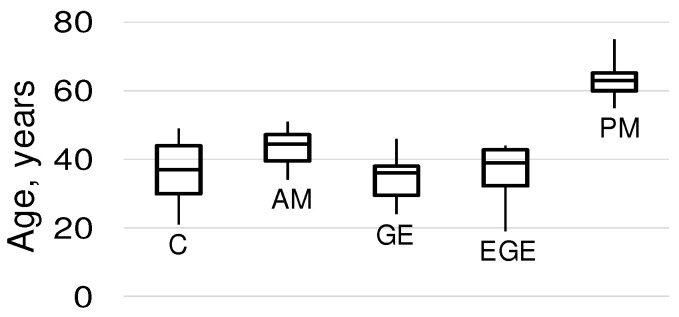
Age of patients. As above, C, control group; AM, adenomyosis; GE, genital endometriosis; EGE, extragenital endometriosis; PM, postmenopause.

In the experimental design, 5 groups were proposed, 4 of which were patients of reproductive age (Figure 4).

Group C, the control group (n = 25), was a control group of patients of reproductive age, without any endometriotic lesions, who were admitted to the hospital for diagnostic hysteroscopy for infertility of unknown etiology or for polyp removal. After separate diagnostic curettage, part of the biomaterial was sent for histological examination (according to the standard protocol for such procedures). The other part (outside the polyp area) was used for our study.

Group AM, the adenomyosis group (n = 24), was a group of patients of reproductive age with adenomyosis, which was established in accordance with ultrasound examinations criteria and during hysteroscopy. Patients were admitted because of either a polyp or menorrhagia. Also, as for group C, the patients underwent separate diagnostic curettage, and part of the obtained biomaterial (in the case of a polyp, outside it) was used for research.

Group GE, the genital endometriosis group (n = 11), was a group of patients of reproductive age with only genital localization of endometriosis (without extragenital foci of endometriosis), endometrioid ovarian cysts (endometrioma), or endometriosis of the fallopian tube (posterior surface of the cervix). Biomaterial of ectopic endometrium for our study was obtained during laparoscopic surgery.

Group EGE, the extragenital endometriosis group (n = 14), was a group of patients of reproductive age who had endometriotic lesions on the pelvic peritoneum, that is, with extragenital localization. Also, as for group GE, biopsies of the ectopic endometrium for research were obtained laparoscopically.

Group PM, the postmenopause group (n = 17), was a group of patients with confirmed menopause (at least 5 years) before admission to the gynecological department for separate diagnostic treatment for a polyp. Patients in this group did not have endometriosis during the entire period of fertility.

Immediately after obtaining the biomaterial intended for our study, part of it was immediately frozen in liquid nitrogen for subsequent RNA isolation and qPR-PCR. The other part was washed in cold 0.9% NaCl, placed in the same pure solution, and transported to the laboratory at a temperature of +4 °C. Just after the sample was delivered to the laboratory, the polarography measurements were carried out. The total time from sample receipt to the start of measurements ranged from 30 to 40 min.

### 4.2. Estimation of the Oxygen Consumption Rate by the Polarography

The final part of cellular metabolism is the production of macroergs such as ATP in the mitochondrial respiratory chain. This occurs with the absorption of O_2_ and the release of CO_2_. To assess mitochondrial effectiveness in our ex vivo study, we used polarography, which allowed us to determine the oxygen consumption rate (OCR).

A part of the biomaterial, which was immediately washed in cold physiological solution after receipt, was transferred to fresh physiological solution after transportation to the laboratory and crushed with scissors. Saponin was added to a final concentration of 10 µg/mL to permeabilize the membranes. Next, it was incubated in a shaker for 10 min, after which the sample was placed in the cuvette of an Oxygraph+ polarograph (Hansatech Instruments Ltd., King’s Lynn, Norfolk, UK) and measurements began by recording the basal oxygen absorption rate, V0.

Further, in accordance with the protocol of Kuznetsov A.V. et al. [50] and our modifications [51], complex I of the respiratory chain substrates was added: 10 mM glutamate + 5 mM malate (#49621 and #374318, respectively, Merck, Burlington, MA, USA). V(I) was recorded. The maximum oxygen consumption rate, Vmax, was recorded after adding 2 mM ADP (#01905, Merck, Burlington, MA, USA). Then, the following inhibitors and substrates were added sequentially: 0.5 µM rotenone (inhibitor of complex I) (#R8875, Merck, Burlington, MA, USA) and 10 mM succinate (substrate of complex II) (#14160, Merck, Burlington, MA, USA). The rate of oxygen uptake V(II) was recorded. Then, 5 µM antimycin A (inhibitor of complex III) (#A8673, Merck, Burlington, MA, USA) and 0.5 mM TMPD + 2 mM ascorbate (artificial substrates of complex IV) (#T7394 and #A7631, respectively, Merck, Burlington, MA, USA) were added, and the rate of oxygen uptake V(IV) was recorded.

To exclude data distortion associated with excessive permeabilization, which could lead to damage to the outer mitochondrial membrane, each sample was tested for its integrity by adding 10 µM cytochrome c (#C3483, Merck, Burlington, MA, USA), and the rate of oxygen uptake was recorded. If this rate was greater than V(IV) by 15%, then such a sample would not be taken into account in the statistics of this group.

After measurement, the tissue was dried and its dry weight was measured. The oxygen consumption rate (OCR) was expressed in pmol O_2_ per ml per min per mg dry mass.

### 4.3. Estimation of the Relative mRNA Content by qRT-PCR

Due to the limited volume of samples obtained during surgery, we were not able to determine the content of proteins involved in metabolism. Therefore, we determined the content of their precursors—mRNA—but for the same reason, only the main components of the respiratory chain and enzymes that catalyze the rate-limiting (irreversible) stages of glycolysis were determined.

Part of the biomaterial, which was frozen, was homogenized and total RNA was isolated using a RNeasy Micro Kit (#74004, Qiagen, Hilden, Germany) according to the manufacturer’s instructions. As a primer for reverse transcription, we used d(T)_15_ and 500 ng of RNA for cDNA synthesis. Specific primers (Table 1) for qPCR were created using Primer3Plus (https://www.primer3plus.com, accessed on 12 June 2022) and Primer-BLAST software (https://www.ncbi.nlm.nih.gov/tools/primer-blast, accessed on 12 June 2022). The quantitative PCR was performed using the Mx300P system (Stratagene, La Jolla, CA, USA) with SYBR green. The expression of target genes was normalized to histone H3 and quantified using the 2^−ΔΔCT^ method [52].

### 4.4. Statistical Analysis

We used the Kolmogorov–Smirnov test of normality for the data distribution. As it was normal, we used two-way ANOVA with the post hoc *t*-test and Bonferroni’s correction for multiple comparisons to estimate the differences between groups, with *p* < 0.05 denoting statistical significance. All the data are presented as the median ± range.

## 5. Conclusions

Summarizing the above, it can be assumed that endometrial biopsies of patients suffering from adenomyosis are characterized by an increase in the oxygen consumption rate due to the terminal complex of the respiratory chain, as well as a decrease in the expression of enzymes that catalyze the irreversible stages of glycolysis.

On the contrary, in biopsies of the ectopic endometrium, endometriomas, and lesions on the pelvic peritoneum, cellular respiration decreased against the background of a decrease in the expression of genes encoding proteins of the respiratory chain complexes. The expression of glycolysis enzymes, in particular pyruvate kinase M1/2, increased, which may be associated with an increase in migration potential and the formation of extragenital foci. Moreover, with extragenital localization, in addition to a decrease, the cellular respiration switched from malate toward succinate utilization, which is typical for hypoxic conditions. In postmenopausal patients, against the background of a decrease in the expression of glycolytic enzymes and respiratory chain proteins, a switch of cellular respiration to the utilization of succinate was also observed; however, this shift was balanced, since the maximum rate of oxygen absorption did not change.

Thus, there are no contradictions between these and previously published results, but the approach to the therapeutic treatment of endometriosis, based on the characteristics of cell metabolism in its foci, should be differentiated depending on the location of the lesion. We can very carefully propose that, in the case of adenomyosis, inhibitors of the terminal stage of cellular respiration could be considered as therapeutic agents (for example, macrolide antibiotics, such as josamycin, etc.), but in the case of endometriomas and extragenital localization, some act as glycolysis inhibitors.

### Limitations of the Study

The main limitation of the study is the fact that we obtained ex vivo data on permeabilized endometrial cells from endometrial lesions of various locations. On the one hand, these data reflect the rate of oxygen absorption by mitochondria, but on the other hand, the possibility of modulation of this process under in vivo conditions cannot be ruled out due to the possible different partial pressures of oxygen in tissues with lesions. Taking this factor into account in subsequent studies will make it possible to draw final conclusions about the changes in oxidative phosphorylation in the cells of endometriosis lesions.

The limited sample volume did not allow us to identify all proteins of interest or to carry out at least two technical replicates of each sample. Therefore, we performed PCR to determine the mRNA content of genes encoding mitochondrial enzymes and enzymes of the rate-limiting (irreversible) stages of glycolysis. Focusing on these studies in the future will provide data for use in the formation of new therapeutic strategies.

## Figures and Tables

**Table 1:** Primer sequences and product sizes.

Gene	Primer Sequence, Forward/Reverse (5′…3′)	Product Size, bp
*CYC1* (Cytochrome c-1)	GAGGTGGAGGTTCAAGACGG/ TAGCTCGCACGATGTAGCTG	160
*COX4I1* (cytochrome c oxidase subunit IV isoform 1)	GCGGCAGAATGTTGGCTAC/ GATGAAGAACATGGCACCGC	341
*ATP5A1* (ATP synthase, H+ transporting, mitochondrial F1 complex, alpha subunit 1)	TCAAAAGACTGGGACTGCTGA/ ATGTACGCGGGCAATACCAT	127
*HK1* (hexokinase 1)	AAGCAGACGCACAACAATGC/ AGGGCCAAGAAGTCACCATTC	92
*HK2* (hexokinase 2)	TGTGGATTCCAGCATTTGCC/ CCTGGCTTTGGTTTCATTGC	70
*PFKL* (phosphofructokinase, liver type)	AAGGGTCAGGTGCAAGAAGTAG/ GATGCGGATGTTCTCCACAATG	129
*PFKM* (phosphofructokinase, muscle type)	CAAAGATGTGACCAAGGCCATG/ TGCGAACCACTCTTAGATACCG	138
*PFKP* (phosphofructokinase, platelet)	TGGACGGAGGCTCAAACATC/ CTGGAAGGAACACCCAGTCC	519
*PKM* (pyruvate kinase M1/2)	GGAAGTGGGCAGCAAGATCT/ GCTCCATCCAGGACTGCATT	564
*H3F3A* (H3 histone, family 3A)	AATCGACCGGTGGTAAAGCA/ GACGCTGGAAGGGAAGTTTG	183

## Data Availability

All data generated or analyzed during this study are included in this article.

## References

[1] Koninckx P.R., Ussia A., Adamyan L., Wattiez A., Gomel V., Martin D.C. (2019). Pathogenesis of endometriosis: The genetic/epigenetic theory. Fertil. Steril..

[2] Flieder D.B., Moran C.A., Travis W.D., Koss M.N., Mark E.J. (1998). Pleuro-pulmonary endometriosis and pulmonary ectopic deciduosis: A clinicopathologic and immunohistochemical study of 10 cases with emphasis on diagnostic pitfalls. Hum. Pathol..

[3] Alzayer H. (2019). Pulmonary endometriosis: A rare cause of hydropneumothorax. Respirol. Case Rep..

[4] Oner A., Karakucuk S., Serin S. (2006). Nasolacrimal endometriosis. A case report. Ophthalmic Res..

[5] Türkçüoğlu I., Türkçüoğlu P., Kurt J., Yildirim H. (2008). Presumed nasolacrimal endometriosis. Ophthalmic Plast. Reconstr. Surg..

[6] Signorile P.G., Viceconte R., Baldi A. (2022). New Insights in Pathogenesis of Endometriosis. Front. Med..

[7] Giudice L.C., Kao L.C. (2004). Endometriosis. Lancet.

[8] Bulun S.E. (2009). Endometriosis. N. Engl. J. Med..

[9] Li J.J., Duan H., Wang S., Sun F.Q., Gan L., Tang Y.Q., Xu Q., Li T.C. (2017). Expression Pattern of G-Protein-Coupled Estrogen Receptor in Myometrium of Uteri with and without Adenomyosis. BioMed Res. Int..

[10] Yilmaz B.D., Bulun S.E. (2019). Endometriosis and nuclear receptors. Hum. Reprod. Update.

[11] Vazquez-Martinez E.R., Bello-Alvarez C., Hermenegildo-Molina A.L., Solis-Paredes M., Parra-Hernandez S., Cruz-Orozco O., Silvestri-Tomassoni J.R., Escobar-Ponce L.F., Hernandez-Lopez L.A., Reyes-Mayoral C. (2020). Expression of Membrane Progesterone Receptors in Eutopic and Ectopic Endometrium of Women with Endometriosis. BioMed Res. Int..

[12] Stephens V.R., Rumph J.T., Ameli S., Bruner-Tran K.L., Osteen K.G. (2022). The Potential Relationship Between Environmental Endocrine Disruptor Exposure and the Development of Endometriosis and Adenomyosis. Front. Physiol..

[13] Signorile P.G., Baldi F., Bussani R., D’Armiento M., De Falco M., Baldi A. (2009). Ectopic endometrium in human foetuses is a common event and sustains the theory of müllerianosis in the pathogenesis of endometriosis, a disease that predisposes to cancer. J. Exp. Clin. Cancer Res..

[14] Signorile P.G., Baldi F., Bussani R., D’Armiento M., De Falco M., Boccellino M., Quagliuolo L., Baldi A. (2010). New evidence of the presence of endometriosis in the human fetus. Reprod. Biomed. Online.

[15] Signorile P.G., Baldi F., Bussani R., Viceconte R., Bulzomi P., D’Armiento M., D’Avino A., Baldi A. (2012). Embryologic origin of endometriosis: Analysis of 101 human female fetuses. J. Cell Physiol..

[16] Laganà A.S., Vitale S.G., Salmeri F.M., Triolo O., Ban Frangež H., Vrtačnik-Bokal E., Stojanovska L., Apostolopoulos V., Granese R., Sofo V. (2017). Unus pro omnibus, omnes pro uno: A novel, evidence-based, unifying theory for the pathogenesis of endometriosis. Med. Hypotheses.

[17] Frankowska K., Dymanowska-Dyjak I., Abramiuk M., Polak G. (2024). The Efficacy and Safety of Transvaginal Ethanol Sclerotherapy in the Treatment of Endometrial Cysts-A Systematic Review. Int. J. Mol. Sci..

[18] Agostini A., De Lapparent T., Collette E., Capelle M., Cravello L., Blanc B. (2007). In situ methotrexate injection for treatment of recurrent endometriotic cysts. Eur. J. Obstet. Gynecol. Reprod. Biol..

[19] Fisch J.D., Sher G. (2004). Sclerotherapy with 5% tetracycline is a simple alternative to potentially complex surgical treatment of ovarian endometriomas before in vitro fertilization. Fertil. Steril..

[20] Gatta G., Parlato V., Di Grezia G., Porto A., Cappabianca S., Grassi R., Rotondo A. (2010). Ultrasound-guided aspiration and ethanol sclerotherapy for treating endometrial cysts. Radiol. Med..

[21] Kim G.H., Kim P.H., Shin J.H., Nam I.C., Chu H.H., Ko H.K. (2022). Ultrasound-guided sclerotherapy for the treatment of ovarian endometrioma: An updated systematic review and meta-analysis. Eur. Radiol..

[22] Schrager S., Yogendran L., Marquez C.M., Sadowski E.A. (2022). Adenomyosis: Diagnosis and Management. Am. Fam. Physician.

[23] Etrusco A., Barra F., Chiantera V., Ferrero S., Bogliolo S., Evangelisti G., Oral E., Pastore M., Izzotti A., Venezia R. (2023). Current Medical Therapy for Adenomyosis: From Bench to Bedside. Drugs.

[24] Kobayashi H., Shigetomi H., Imanaka S. (2021). Nonhormonal therapy for endometriosis based on energy metabolism regulation. Reprod. Fertil..

[25] Wu M.H., Hsiao K.Y., Tsai S.J. (2019). Hypoxia: The force of endometriosis. J. Obstet. Gynaecol. Res..

[26] Atkins H.M., Bharadwaj M.S., O’Brien Cox A., Furdui C.M., Appt S.E., Caudell D.L. (2019). Endometrium and endometriosis tissue mitochondrial energy metabolism in a nonhuman primate model. Reprod. Biol. Endocrinol..

[27] Horne A.W., Ahmad S.F., Carter R., Simitsidellis I., Greaves E., Hogg C., Morton N.M., Saunders P.T.K. (2019). Repurposing dichloroacetate for the treatment of women with endometriosis. Proc. Natl. Acad. Sci. USA.

[28] Monsivais D., Dyson M.T., Yin P., Coon J.S., Navarro A., Feng G., Malpani S.S., Ono M., Ercan C.M., Wei J.J. (2014). ERβ- and prostaglandin E2-regulated pathways integrate cell proliferation via Ras-like and estrogen-regulated growth inhibitor in endometriosis. Mol. Endocrinol..

[29] Monsivais D., Dyson M.T., Yin P., Navarro A., Coon J.S.T., Pavone M.E., Bulun S.E. (2016). Estrogen receptor β regulates endometriotic cell survival through serum and glucocorticoid-regulated kinase activation. Fertil. Steril..

[30] Li J., Yanyan M., Mu L., Chen X., Zheng W. (2019). The expression of Bcl-2 in adenomyosis and its effect on proliferation, migration, and apoptosis of endometrial stromal cells. Pathol. Res. Pract..

[31] Brunelle J.K., Letai A. (2009). Control of mitochondrial apoptosis by the Bcl-2 family. J. Cell Sci..

[32] Kagan V.E., Borisenko G.G., Tyurina Y.Y., Tyurin V.A., Jiang J., Potapovich A.I., Kini V., Amoscato A.A., Fujii Y. (2004). Oxidative lipidomics of apoptosis: Redox catalytic interactions of cytochrome c with cardiolipin and phosphatidylserine. Free Radic. Biol. Med..

[33] Lambert A.J., Brand M.D. (2009). Reactive oxygen species production by mitochondria. Methods Mol. Biol..

[34] Liu F., He L., Liu Y., Shi Y., Du H. (2013). The expression and role of oxidative stress markers in the serum and follicular fluid of patients with endometriosis. Clin. Exp. Obstet. Gynecol..

[35] Bamm V.V., Henein M.E.L., Sproul S.L.J., Lanthier D.K., Harauz G. (2017). Potential role of ferric hemoglobin in MS pathogenesis: Effects of oxidative stress and extracellular methemoglobin or its degradation products on myelin components. Free Radic. Biol. Med..

[36] Toniyan K.A., Gorbacheva E.Y., Golubkova M.A., Povorova V.V., Boyarintsev V.V., Ogneva I.V. (2023). Cytochrome-c-oxidase and ATP synthase content increases in the endometrium of the patients with adenomyosis. Mol. Biol. Rep..

[37] Kapur A., Ayuso J.M., Rehman S., Kumari S., Felder M., Stenerson Z., Skala M.C., Beebe D., Barroilhet L., Patankar M.S. (2023). Oxidative phosphorylation inhibitors inhibit proliferation of endometriosis cells. Reproduction.

[38] Lamceva J., Uljanovs R., Strumfa I. (2023). The Main Theories on the Pathogenesis of Endometriosis. Int. J. Mol. Sci..

[39] Kobayashi H., Kimura M., Maruyama S., Nagayasu M., Imanaka S. (2021). Revisiting estrogen-dependent signaling pathways in endometriosis: Potential targets for non-hormonal therapeutics. Eur. J. Obstet. Gynecol. Reprod. Biol..

[40] Sahni P.V., Zhang J., Sosunov S., Galkin A., Niatsetskaya Z., Starkov A., Brookes P.S., Ten V.S. (2018). Krebs cycle metabolites and preferential succinate oxidation following neonatal hypoxic-ischemic brain injury in mice. Pediatr. Res..

[41] Hou S., Lei S., Peng H., Weng L., Lv S., Li M., Zhao D. (2022). Downregulating HK2 inhibits proliferation of endometrial stromal cells through a noncanonical pathway involving phosphorylation of signal transducer and activator of transcription 1 in endometriosis. Biol. Reprod..

[42] Bramer S.A., Macedo A., Klein C. (2017). Hexokinase 2 drives glycogen accumulation in equine endometrium at day 12 of diestrus and pregnancy. Reprod. Biol. Endocrinol..

[43] Christofk H.R., Vander Heiden M.G., Harris M.H., Ramanathan A., Gerszten R.E., Wei R., Fleming M.D., Schreiber S.L., Cantley L.C. (2008). The M2 splice isoform of pyruvate kinase is important for cancer metabolism and tumour growth. Nature.

[44] Atas E., Oberhuber M., Kenner L. (2020). The Implications of PDK1-4 on Tumor Energy Metabolism, Aggressiveness and Therapy Resistance. Front. Oncol..

[45] Hamza A., Cho J.Y., Cap K.C., Hossain A.J., Kim J.G., Park J.B. (2023). Extracellular pyruvate kinase M2 induces cell migration through p-Tyr42 RhoA-mediated superoxide generation and epithelial-mesenchymal transition. Free Radic. Biol. Med..

[46] Toniyan K.A., Povorova V.V., Gorbacheva E.Y., Boyarintsev V.V., Ogneva I.V. (2021). Organization of the Cytoskeleton in Ectopic Foci of the Endometrium with Rare Localization. Biomedicines.

[47] Liao T.L., Tzeng C.R., Yu C.L., Wang Y.P., Kao S.H. (2015). Estrogen receptor-? in mitochondria: Implications for mitochondrial bioenergetics and tumorigenesis. Ann. N. Y. Acad. Sci..

[48] Wang T., Zhang J., Hu M., Zhang Y., Cui P., Li X., Li J., Vestin E., Brännström M., Shao L.R. (2019). Differential Expression Patterns of Glycolytic Enzymes and Mitochondria-Dependent Apoptosis in PCOS Patients with Endometrial Hyperplasia, an Early Hallmark of Endometrial Cancer, In Vivo and the Impact of Metformin In Vitro. Int. J. Biol. Sci..

[49] Kong A., Johnson N., Kitchener H.C., Lawrie T.A. (2012). Adjuvant radiotherapy for stage I endometrial cancer. Cochrane Database Syst. Rev..

[50] Kuznetsov A.V., Veksler V., Gellerich F.N., Saks V., Margreiter R., Kunz W.S. (2008). Analysis of mitochondrial function in situ in permeabilized muscle fibers, tissues and cells. Nat. Protoc..

[51] Ogneva I.V., Usik M.A., Burtseva M.V., Biryukov N.S., Zhdankina Y.S., Sychev V.N., Orlov O.I. (2020). Drosophila melanogaster Sperm under Simulated Microgravity and a Hypomagnetic Field: Motility and Cell Respiration. Int. J. Mol. Sci..

[52] Livak K.J., Schmittgen T.D. (2001). Analysis of relative gene expression data using real-time quantitative PCR and the 2(-Delta Delta C(T)) Method. Methods.

